# A questionnaire for determining prevalence of diabetes related foot disease (Q-DFD): construction and validation

**DOI:** 10.1186/1757-1146-2-34

**Published:** 2009-11-25

**Authors:** Shan M Bergin, Caroline A Brand, Peter G Colman, Donald A Campbell

**Affiliations:** 1 Monash Institute of Health Services Research, Monash University, Kanooka Gve Clayton, Melbourne, Australia; 2 Department of Diabetes and Endocrinology, The Royal Melbourne Hospital, Gratten St, Parkville, Melbourne, Australia; 3 Clinical Epidemiology and Health Service Evaluation Unit, The Royal Melbourne, Hospital, Gratten St, Parkville, Melbourne, Australia; 4 Centre for Research Excellence in Patient Safety, Monash University, Melbourne, Australia; 5 Department of General Medicine, Monash University, Wellington Rd, Clayton, Melbourne, Australia

## Abstract

**Background:**

Community based prevalence for diabetes related foot disease (DRFD) has been poorly quantified in Australian populations. The aim of this study was to develop and validate a survey tool to facilitate collection of community based prevalence data for individuals with DRFD via telephone interview.

**Methods:**

Agreed components of DRFD were identified through an electronic literature search. Expert feedback and feedback from a population based construction sample were sought on the initial draft. Survey reliability was tested using a cohort recruited through a general practice, a hospital outpatient clinic and an outpatient podiatry clinic. Level of agreement between survey findings and either medical record or clinical assessment was evaluated.

**Results:**

The Questionnaire for Diabetes Related Foot Disease (Q-DFD) comprised 12 questions aimed at determining presence of peripheral sensory neuropathy (PN) and peripheral vascular disease (PVD), based on self report of symptoms and/or clinical history, and self report of foot ulceration, amputation and foot deformity. Survey results for 38 from 46 participants demonstrated agreement with either clinical assessment or medical record (kappa 0.65, sensitivity 89.0%, and specificity 77.8%). Correlation for individual survey components was moderate to excellent. Inter and intrarater reliability and test re-test reliability was moderate to high for all survey domains.

**Conclusion:**

The development of the Q-DFD provides an opportunity for ongoing collection of prevalence estimates for DRFD across Australia.

## Background

Diabetes related foot disease (DRFD) describes a number of complications of diabetes that can occur simultaneously or in isolation. Peripheral neuropathy (PN), peripheral vascular disease (PVD), foot ulceration and amputation contribute significantly to the high rates of morbidity and mortality affecting individuals with diabetes [1-6]. Despite the burden of foot disease on both the individual and the health care system, little research has been conducted in order to determine its prevalence in the community in Australia. The paucity of Australian data describing the prevalence of DRFD makes future planning and policy direction for health services extremely difficult. The scope and geographical distribution of chronic disease, including DRFD, are essential pre-requisites for ensuring targeted health care resources are available where and when they are needed. Mapping changes in disease prevalence over time is also required in order to support the planning and distribution of health services into the future. This is especially important given that required changes to service provision are most likely to be in response to increasing, rather than decreasing disease prevalence.

Establishing the true epidemiology of DRFD is complex, resulting in wide variation in reported prevalence estimates [7-11]. Differences in study methodologies including methods for population selection and sampling are likely to have the greatest impact on prevalence estimates. Samples derived from hospital based outpatient clinics are more likely to be selected due to their availability and the ease with which they can be comprehensively studied [12]. However, it is well documented that such samples tend to yield biased estimates for disease prevalence when compared with community based samples and complications present tend to be more advanced in terms of severity when compared with community based populations [12,13]. A population based sampling strategy is therefore preferred in order to generate more accurate estimates of community based prevalence of DRFD [12,14,15].

In Australia, the identification of a reference population and appropriate sampling and recruitment strategy for use in determining the prevalence of DRFD is further complicated by the geographical dispersion of the population. Whilst clinical examination is arguably the gold standard for identifying individuals with DRFD, bringing together suitably qualified clinical examiners and a representative sample of individuals which includes those living outside major city centres, is both time consuming and costly. Therefore, a valid and reliable survey instrument that is easy to administer, would be a valuable and cost effective means of identifying those persons with DRFD in the community and could potentially be used for both epidemiological surveys and clinical screening purposes.

The aim of this study was to develop and evaluate such a survey tool, with the intention that it be used to identify community based individuals with DRFD via telephone interview, without the need for clinical examination. The development of a survey tool would allow for prevalence data to be collected from a representative sample of the Australian population with the advantage of reduced time and cost. Furthermore, the availability of a valid and reliable tool would facilitate ongoing and more widespread collection of prevalence data for DRFD in Australia. Clearly, an important use of this data would be to identify where those affected by DRFD are located and to assist in the future planning and allocation of health care services.

## Methods

**Figure 1:**
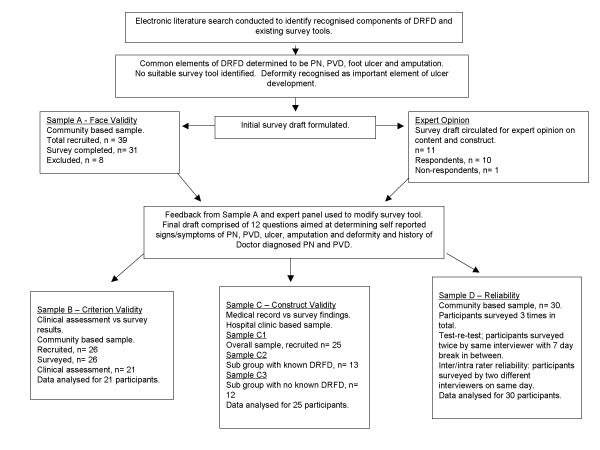
**Methodological steps used for survey development**. This flowchart depicts the steps taken to develop and validate the survey tool. It incorporates the steps used to determine face, criterion and construct validity as well as survey reliability. Overall 107 study participants and eleven 'experts' were used to confirm that the survey was both valid and reliable.

Ethical approval was granted by The Melbourne Health Human Research and Ethics Committee, The Monash University Standing Committee on Ethics in Research Involving Humans and The Alfred Human Research Ethics Committee. The questionnaire development is also presented diagrammatically in Figure 1.

### Development of the survey tool

An electronic literature search was conducted by the primary researcher (SB) in order to identify the consensus components of DRFD and any survey tools already in use. The search was made of MEDLINE (1950 - July, week 4, 2006) and CINAHL (1950 - July 2006) and also included the websites of local and international diabetes organisations.

Whilst the literature search identified two surveys that were used to identify the presence or absence of PN (sensory, motor and autonomic) and PVD respectively, it failed to identify any existing survey tools that encompassed all aspects of DRFD within the one tool. As a result, development of a new survey tool was undertaken.

### Survey validity

An initial draft of the survey tool was compiled using results from the electronic literature search and forwarded to eleven individuals with recognised expertise in the areas of diabetes, assessment and management of the diabetic foot, epidemiology and survey design and application. The 11 experts were selected based on one or more of the following: known reputation in their field, number of publications (lead or co-author) relating to their area of expertise or years of clinical practice in diabetes and/or foot complications. This group of experts was invited to provide feedback on the survey content and construct. Face validity, or the appearance that the survey is testing what it is supposed to, was further determined using a community based construction sample (Sample A). The community based sample was recruited from an advertisement placed in a diabetes consumer magazine produced by Diabetes Australia, the national diabetes organisation. The advertisement made no specific reference to foot complications in order to reduce response bias in favour of those with complications. Respondents were required to be 45 years of age or over, be permanent residents of Victoria, be diagnosed with type 1 or type 2 diabetes and be sufficiently competent in English to complete a survey interview over the phone. Those meeting the inclusion criteria were invited to call a specified number and leave their name and contact details and to nominate a preferred day and time to be contacted.

Having completed the survey, consenting participants were asked to provide feedback on the acceptability of the survey instrument, including language used, length of the survey and survey content. This sample was also used to record data such as average length of time required to complete a survey interview and number of calls required per person to complete a survey.

### Criterion and construct validity

The degree to which the survey identified patients with no known DRFD and identified those with existing DRFD was tested using community based (Sample B) and hospital clinic based (Sample C) patient cohorts. The same inclusion criteria applied. The community based Sample B was recruited via advertisements placed in suburban newspapers and through a General Practice located in North East Melbourne. Participants were invited to complete the survey via telephone and then attend for a clinical assessment. Consent for conduct of the survey was assumed if the survey was completed at the time of the call and written consent was obtained at the time of clinical assessment.

The survey and the clinical assessments were performed independently of each other with the survey administered by an experienced research nurse and the assessment conducted by a podiatrist. Survey results were not made available to the podiatrist prior to conduct of the clinical assessments. The clinical examination included assessment for peripheral sensory neuropathy using a 10 g Semmes Weinstein Monofilament (applied to the apex of the 1^st^, 3^rd ^and 5^th ^toes and the plantar aspect of the 1^st ^and 5^th ^metatarsophalangeal joints) and assessment for vasculopathy by determining bilateral Ankle Brachial Indices (using an 8 mHz hand held Doppler, standard blood pressure cuff and sphygmomanometer) and manual palpation of pedal pulses. The presence of foot deformity or pressure areas was recorded, as was history of amputation and past and present history of ulceration. Components for clinical assessment were based on current literature and best practice recommendations for clinical evaluation [16-18].

The clinic based Sample C was recruited from consecutive attendees at a diabetes outpatient clinic at a major tertiary hospital as they attended for a routine appointment. This sample included individuals with known DRFD and individuals with no known foot complications; each was nominated as meeting the inclusion criteria by their Endocrinologist, and was then invited to participate by the researcher. Individuals were asked to provide contact details so that an independent interviewer could call them in one week's time in order to conduct the survey over the telephone. At the time of phone contact verbal consent was re-confirmed with these individuals prior to the survey being undertaken to ensure that each was given the opportunity to withdraw consent given at the time of recruitment. Individual survey results were then compared with medical records, which were searched for any recorded evidence of diabetes related foot complications in particular PN, PVD, ulceration and amputation. Participants provided written consent for review of their medical records.

### Survey reliability

Interrater, intrarater and test-retest reliability was assessed using a convenience sample from a community health centre podiatry department (Sample D). The same inclusion criteria used for previous samples was applied. Clinic staff from the podiatry department were educated regarding the inclusion criteria, and the requirements for participation in the study. Staff then assisted with recruitment of potential participants as they attended for routine appointments. Written consent was obtained from all participants at the time of recruitment. Participants were required to complete the survey via telephone interview on three separate occasions with the initial two surveys administered on the same day by two independent interviewers who were blinded to each others survey findings (interrater reliability). The third survey was conducted seven days later by one of the initial interviewers in order to assess intrarater and test-retest reliability.

### Statistical analysis

Prevalence rates for Samples B and C were calculated as absolute frequencies and are reported as overall percentages. Agreement between survey results and clinical assessment for Sample B and survey findings and medical record for Sample C was analysed and reported using reliability coefficient kappa (where perfect agreement equals +1.00). Sensitivity and specificity are reported for samples B and C as are likelihood ratios (LR+ and LR-), which combine the information provided by sensitivity and specificity, to give an indication of how much the odds of disease change based on a positive or negative result. Inter and intrarater, and test-retest reliability was evaluated for Sample D with overall correlation reported using kappa statistic. Prevalence rates were not calculated for this cohort.

## Results

Search results established the most commonly occurring diabetes related lower limb and foot disorders to be peripheral neuropathy, peripheral vascular disease, ulceration and lower limb and foot amputation. Consequently, survey domains were constructed that dealt with each of these components. Whilst foot deformity was not recognised as a true component of DRFD it was widely recognised as playing a significant role in the development of foot ulcers and was therefore included as a survey domain.

### Face validity - expert and patient feedback

Feedback from 10 out of 11 experts invited to review the initial survey draft confirmed all survey domains were appropriate and inclusive; no response was received from one individual invited to participate in this aspect of the study despite an invitation to participate being sent on three separate occasions. Suggestions regarding the survey format and language were used to modify the original draft.

Of the 39 participants who comprised construction Sample A, 31 (79.5%) completed the survey via telephone interview. The remaining eight were excluded as they either withdrew consent at the time of contact (n = 2) or were unavailable or could not be reached during the survey period (n = 5). One phone number had been disconnected. Participant characteristics are shown in Table 1 and prevalence findings for this group are shown in Table 2. Ninety-three calls were required to complete the 39 surveys with an average of 2 calls made per person and the average call time was six minutes (range 2-12 minutes). One hundred percent of responding participants reported satisfaction with both the survey content, the length of time it took to complete the survey and the language used within the survey. No modifications were made to the survey based on feedback from this sample.

**Table 1:** Descriptive data for all patient cohorts used to establish validity and reliability of the survey tool.

Sample						
	A	B	C1	C2	C3	D
Total participants	31	21	25	13	12	30

Mean Age (years)	64.0 (range 45-80)	67.1 (range 45-83)	64.7 (range 45-77)	68.0 (range57-77)	61.0 (range 45-76)	

Mean diabetes Duration (years)	10.2 (range 1-55)	13.7 (range 1-36)	19.9 (range 1-54)	25.7 (range 5-54)	13.5 (range 2-37)	

Male	15 (48.0%)	10 (48.0%)	15 (60.0%)	10 (77.0%)	5 (42.0%)	17 (57.0%)

Female	16 (52.0%)	11 (52.0%)	10 (40.0%)	3 (23.0%)	7 (58.0%)	13 (43.0%)

C1 - clinic based cohort, survey Vs medical record, C2 - clinic based cohort, survey Vs medical record, with known foot complications

C3 - clinic based cohort, survey Vs medical record, with no known foot complications

Sample A was used to determine face validity, Sample B was used to determine criterion validity and

Sample C was used to determine construct validity. Sample D was used to establish test re-test and inter and intrarater reliability.

**Table 2:** Prevalence findings for individual components of DRFD for each patient cohort.

Prevalence (%)					
	Peripheral Neuropathy	Peripheral Vasculopathy	Ulceration	Amputation	Deformity
Sample A	29.0	16.0	6.0	0.0	71.0
Sample B	38.0	14.0	0.0	0.0	48.0
Sample C*	42.0	52.0	44.0	24.0	-
Sample C**	77.0	77.0	77.0	46.0	-
Sample C***	33.0	25.0	8.0	0.0	-

Sample C* - Overall prevalence for all of Sample, Sample C** - Overall prevalence for Sample C sub group with known foot complications

Sample C*** - Overall prevalence for Sample C sub group with no known foot complications

This data is calculated as absolute frequencies and is reported as percentage total of each cohort. All percentage figures have been rounded up to whole numbers. No deformity data is reported for Sample C as this information was not routinely recorded in the medical record.

### The survey tool

The final survey comprised 12 questions aimed at identifying the presence or absence of clinically diagnosed sensory PN or PVD and/or the presence or absence of self reported signs and symptoms for sensory PN, PVD, foot ulcers, amputation and foot deformity. The PN domain was confined to determining presence of sensory neuropathy given the significant role this plays in the development of foot ulcers.

Components from two previously validated survey tools, The Neuropathy Symptom Score (NSS) and The Edinburgh Claudication Questionnaire (ECQ), were used to construct the diagnostic domains of the survey that dealt with sensory PN and PVD [15,19,20]. These survey questions were based on the most commonly occurring symptoms for these two pathologies and required dichotomous 'yes/no' responses [19,20]. For sensory PN and/or PVD to be identified based on symptomology, one or more of the nominated symptoms must have been present for a minimum of one month, have occurred consistently over that time period and could not potentially be related to any other pathology. The symptoms used in order to diagnose sensory neuropathy were burning, tingling, numbness, pins and needles and tightness, whilst the PVD symptoms included claudication and rest pain. One open ended question in each domain allowed participants to elaborate on the timing of symptoms, what relief they sought for their symptoms and how effective these interventions were and what possible causes, other than diabetes, could be responsible for their symptoms. Where any doubt existed over the cause of reported symptoms, a negative diagnosis was made.

A series of questions were also included that aimed to identify sensory PN and PVD that had previously been clinically diagnosed by a healthcare professional. These questions were asked in three different ways, to accommodate differences in language used by the wide variety of health care professionals who may potentially diagnose these pathologies, and to accommodate different levels of understanding of participants (Table 3). As part of this domain a single question was included regarding history of surgical intervention for PVD.

**Table 3:** Summary statistics for assessment of criterion and construct validity.

	Kappa	Sensitivity %	Specificity %	LR+	LR-
Samples B and C combined (any diagnosis of DRFD)	0.64	85.0 (63.9, 94.8)	79.2 (59.5, 90.8)	4.1 (2.7, 6.2)	0.19 (0.09, 0.37)

Samples B and C combined (all components of DRFD)					
					
PN	0.70	85.7 (65.4, 95.0)	84.6 (66.5, 94.0)	5.6 (3.4, 9.3)	0.2 (0.09, 0.32)
PVD	0.60	83.3 (55.2, 95.3)	83.3 (68.1, 92.1)	5.0 (3.5, 7.2)	0.2 (0.07, 0.54)
Ulcer	0.90	91.7 (64.6, 98.5)	97.2 (85.8, 99.5)	33.0 (4.6, 238.0)	0.08 (0.01, 0.61)
Amputation	0.83	85.7 (48.7, 88.7)	97.5 (87.1, 99.6)	34.3 (4.6, 257.0)	0.14 (0.02, 1.04)
Deformity **					

Sample B (any diagnosis of DRFD)	0.43	60.0 (23.1, 88.2)	84.6 (57.8, 95.7)	3.9 (0.95, 16.1)	0.47 (0.17, 1.3)

Sample B (all components of DRFD)					
					
PN	0.57	75.0 (40.9, 92.9)	84.6 (57.8, 95.7)	4.9 (1.6, 14.5)	0.29 (0.12, 0.8)
PVD	0.77	66.8 (20. 8, 93.9)	94.8 (75.4, 99.1)	12.7 (1.1, 147)	0.35 (0.05, 2.5)
Deformity	0.72	90.9 (62.3, 98.4)	72.7 (43.4, 90.3)	3.3 (1.7, 6.5)	0.13 (0.02, 1.0)

Sample C (any diagnosis of DRFD)	0.67	93.0 (70.2, 98.8)	72.7 (43.4, 90.3)	3.4 (1.8, 6.6)	0.09 (0.01, 0.71)

Sample C (all components of DRFD)					
					
PN	0.84	92.3 (66.7, 98.6)	84.6 (57.8, 95.7)	6.0 (2.2, 16.2)	0.09 (0.01, 0.7)
PVD	0.61	88.9 (56.5, 98.0)	70.6 (46.9, 86.7)	3.0 (2.0, 4.6)	0.16 (0.02, 1.2)
Ulcer	1.00	91.7 (64.6, 98.5)	93.3 (70.2, 98.8)	13.8 (1.9, 99.0)	0.09 (0.01, 0.6)
Amputation	0.90	85.7 (48.7, 97.4)	94.7 (75.4, 99.1)	16.3 (2.2, 122.0)	0.14 (0.02, 1.0)

Sample C (complications group, all components of DRFD)					
					
PN	0.75	90.9 (62.3, 98.4)	66.7 (20.8, 93.9)	2.7 (0.38, 19.8)	0.14 (0.01, 1.6)
PVD	0.41	85.7 (48.7, 97.4)	42.9 (15.8, 75.0)	1.5 (0.9, 2.6)	0.33 (0.02, 5.7)
Ulcer	1.00	90.9 (62.3, 98.4)	75.0 (30.1, 95.4)	3.6 (0.5, 26.3)	0.12 (0.01, 1.1)
Amputation	0.85	85.7 (48.7, 97.4)	85.7 (48.7, 97.4)	6.0 (0.8, 45.0)	0.17 (0.02, 1.2)

Sample C (no complications group, all components of DRFD)					
					
PN	0.75	66.7 (20.8, 93.9)	90.0 (59.6, 98.2)	6.7 (0.6, 77.3)	0.4 (0.05, 2.7)
PVD	0.75	66.7 (20.8, 93.9)	90.0 (59.6, 98.2)	6.7 (0.6, 77.3)	0.4 (0.05, 2.7)
Ulcer	1.00	50.0 (9.5, 90.6)	91.7 (64.6, 98.5)	6.0 (0.12, 302.0)	0.55 (0.07, 3.9)
Amputation	1.00	50.0 (9.5, 90.6)	92.3 (66.7, 98.6)	6.5 (0.13, 328.0)	0.54 (0.07, 3.9)

CI set at 95%, LR+ = positive likelihood ratio, LR- = negative likelihood ratio. No analysis for sample B for ulcer and amputation as no respondent reported either component. ** no combined analysis completed due to lack of deformity data in Sample C

For Sample B (n = 21) correlation between survey findings and clinical assessment were analysed and for Sample C (n = 25) correlation between survey findings and medical record were analysed. Analysis was also undertaken for two subgroups of Sample C, one with known foot complications (n = 13) and one with no known foot complications (n = 12). Findings are reported for any diagnosis of DRFD (where any one of PN, PVD, ulcer, amputation or deformity were identified) and for the diagnosis of individual DRFD components.

Questions relating to the remaining components of DRFD, foot ulcer and amputation, were included, as were questions regarding commonly known foot deformities such as hammer or clawed toes, Hallux Abducto Valgus (bunions) and corns and callus. Each of these questions required dichotomous 'yes/no' responses and information pertaining to the timing of foot ulcers (ie were ulcers current or resolved) and the timing and level of amputation (foot amputation, below knee amputation [BKA] and above knee amputation [AKA]) were also included.

To facilitate accurate reporting of presence or absence of amputation and ulceration appropriate definitions were provided to all survey participants if required. Definitions were also provided for all deformities listed as part of the survey and each was described in simple terms to maximise the likelihood of accurate reporting. Questions pertaining to diabetes history and demographics were also included.

### Criterion and construct validity

Survey results for 46 participants were evaluated against the gold standard of either clinical assessment (Sample B, n = 21) or medical record (Sample C, n = 25). Participant characteristics are noted in Table 1. A further five participants from Sample B were surveyed but failed to attend for clinical examination and were therefore excluded from any analysis. Survey responses for 38 out of 46 participants demonstrated agreement with either clinical assessment or medical record for an overall diagnosis of DRFD, where any one of sensory PN, PVD, ulcer or amputation were identified (kappa 0.65 [0.37, 0.94], sensitivity 89.0% [68.6, 97.1], specificity 77.8% [59.2, 89.4]). Deformity was not included in this analysis as it was not routinely recorded in the medical histories reviewed for participants from Sample C; therefore not enough data was available for comparison with survey findings. Summary data for levels of agreement for individual components of the survey and for Samples B and C combined and individually are provided in Table 4. Survey prevalence findings for sensory PN, PVD, ulceration, amputation and deformity can be seen in Table 2.

**Table 4:** Comparison of prevalence rates for PN, PVD and foot ulcer between this study and international studies.

First author	Study year	Study location	Diabetes prevalence	Prevalence of PVD	Prevalence of PN	Prevalence of foot ulceration
Bergin (current study)	2008	Australia	7.0%	16.0%	37.0%	4.5%

Manes	2002	Greece	8.7%	12.7%	33.5%	4.7%

Nielsen	1998	Saudi Arabia	23.0%	NR*	38.0%	4.7%

Kastenbauer	2004	Austria	5.0%	37.5%	NR	NR

Rhee	2007	Asia	6.8%**	17.7%	NR	NR

Hirsch	2001	United States	6.8%	19.6%	NR	NR

Al-Mahroos	2007	Bahrain	18.0%	NR	NR	5.9%

*NR = not reported.

Diabetes prevalence data is sourced from the World Health Organisation and is compiled using local epidemiological studies and surveys. Amputation is not included here as most international studies report findings as per capita or incidence rates not as prevalence rates. Deformity is not included here as no local or international deformity data could be identified for use as a comparison. ** Prevalence data for Asia is reported as a mean value given that this study was conducted across 7 Asian countries.

### Survey reliability

Thirty patients attending a community health podiatry clinic (Sample D) were recruited as they attended for their routine podiatry appointments. All 30 patients completed all three survey interviews. Patient characteristics are seen in Table 1.

Interrater reliability for a diagnosis of DRFD (where any one of sensory PN, PVD, ulcer or amputation was identified) was excellent (kappa = 1.00). Interrater reliability for individual components of the survey were moderate to high for all domains except for deformity; sensory PN (kappa = 0.52), PVD (kappa = 0.67), ulcer (kappa = 1.00), amputation (kappa = 0.72), deformity (kappa = 0.37). Intrarater and test-retest reliability were also moderate to high for all survey domains with identification of any component of DRFD achieving a kappa score of 0.53 and the domains of sensory PN, PVD, ulcer and amputation achieving scores of 0.71, 0.52, 1.0 and 1.0 respectively. Deformity was the least reliable survey domain with a kappa score of 0.42.

## Discussion

Whilst it is widely accepted that clinical examination is the gold standard for determining presence or absence of disease, it is also acknowledged that in the conduct of large community based epidemiological studies, this may not be feasible [14]. With this in mind, alternative methods are required in order to ensure ongoing and widespread collection of data that provides a reliable estimate of disease prevalence. The Questionnaire for Diabetes Related Foot Disease (Q-DFD), which combines previously validated diagnostic survey questions with additional non-diagnostic components, provides such an alternative. The survey tool, which unlike previously developed tools encompasses all aspects of DRFD, has proven to be both valid and reliable for all cohorts studied here.

Appropriate assessment for validity is important to ensure the accuracy of any diagnostic survey. In this instance, face (Sample A), construct (Sample C) and most importantly, criterion validity (Sample B), were all measured using appropriately selected study samples of similar size. Whilst face validity is viewed as the least robust of all measures of validity, it is an important part of the development process for any measurement tool and is useful for ensuring a survey yields the most relevant information. In order to enhance the strength of the Q-DFD, construct and criterion validity were examined alongside face validity. Construct validity is more robust than face validity and demonstrates a survey's ability to identify differences between two groups. Sample C compared survey findings for those with known foot complications to those with no known foot complications. The overall kappa score of 0.67 for any diagnosis of DRFD demonstrates a substantial, and therefore acceptable, level of agreement.

Comparison with a known 'gold standard' is considered the best way to determine the validity of a diagnostic survey. With this in mind the Q-DFD was tested against best practice clinical examination and the sensitivity and specificity of the findings reported (Sample B).

The lowest overall level of agreement for Sample B (kappa = 0.43) indicates a moderate level of agreement for an overall diagnosis of DRFD. This cohort also demonstrated the lowest sensitivity (60.0%) meaning that prevalence estimates may be underestimated based on findings for this group alone. Overall agreement increased to 'substantial' when Samples B and C were combined (kappa = 0.64) with a corresponding increase noted in sensitivity and a small decrease seen in specificity. Level of agreement for individual components of the survey for both Samples B and C were moderate to excellent for all aspects when analysed alone and in combination. The increased agreement noted when the two samples were analysed together is not unexpected given the overall increased prevalence of complications within Sample C, due to the inclusion of a cohort with known DRFD. The slightly lower correlation score for Sample B may also be a function of sample size, an assumption supported by the narrow confidence intervals for LR- and the fact that the lower limit of the CI for the LR+ is less than 1.00 (0.95). Overall, the equal weighting given to face, construct and criterion validity has ensured a robust evaluation of the Q-DFD.

To demonstrate variation with use of the Q-DFD is within reasonable limits, inter and intra rater reliability and test-retest reliability was also conducted. Inter and intrarater reliability was moderate to high for the diagnostic domains of PVD and sensory PN indicating the surveys accuracy regardless of who is conducting the interview. Reliability scores for the deformity domain were not as high indicating a reduced correlation between self report of foot deformity/pressure and clinical presentation. This was thought to reflect differences in podiatrist and participant perceptions of what constitutes a pressure area. The podiatrist conducting the clinical examination may only have recorded the presence of pressure areas significant enough to warrant clinical intervention, whereas it was thought that participants were perhaps reporting less significant pressure areas that would not require clinical care. It may therefore be prudent to clarify with participants of future surveys whether they require any treatment or care for their pressure areas or any deformity noted. Further to this, accurate identification of pressure areas may be influenced by the presence of sensory PN, with reduced sensory feedback increasing the potential for such trauma to go unnoticed.

The less than perfect interrater reliability kappa score of 0.72 for amputation was found to be a result of errors identified within the medical records of some participants, whereby participants noted they had undergone amputation however none was noted in the medical record. With these errors corrected, the kappa would in fact have been 1.00 reflecting an excellent degree of reliability.

Prevalence ratings calculated appear to be consistent with international reports of community prevalence for all components of DRFD [21-26]. Deformity, which is not routinely included in community based studies, was common within this cohort, but there is little data available for comparison.

The survey findings from this study do differ somewhat from the findings of the 2003 AusDiab foot complications study, in particular for prevalence of PN [12]. Using clinical examination to determine presence or absence of PN and PVD, AusDiab reported prevalence for PN to be just 13.1% in those with known diabetes. This is significantly lower than the community prevalence rates for PN from the current study which were 29.0% (Sample A), 38.0% (Sample B) and 42.0% (Sample C). The AusDiab findings are also significantly lower than those reported in other international studies. Findings for prevalence of PVD and ulceration were more consistent across studies, with AusDiab reporting prevalence rates of 13.9% for PVD and 3.0% for ulceration compared with an average prevalence rate for PVD of 15.0% and an average prevalence rate for ulceration of 4.5% from this study. As acknowledged by the authors of AusDiab, the low prevalence for PN prevalence is a possible function of sample selection used for their study and may also relate to the short mean disease duration (2.5 years) recorded for their cohort. Unfortunately, there is no other Australian community prevalence data for DRFD with which to compare our findings. Similarly, it is difficult to find another survey tool with which to compare the Q-DFD, because no other survey tool to date includes all aspects of DRFD in the one survey.

Whilst this study indicates the reliability of the Q-DFD is sufficient to warrant ongoing use, the limitations of using survey tools alone to determine presence or absence of disease must be acknowledged. In particular, the impact accuracy of self reporting of disease symptoms can have on overall findings and the potential for over estimation of disease prevalence is a consideration. However, results from this study do not indicate an overestimation of disease prevalence using the Q-DFD, when findings are compared to similar population based studies reported from other countries [21-26]. Further to this, any over estimation of disease prevalence that does not result in significant cost or harm to the relevant patient group or the health system, makes slight variation from true estimates acceptable.

What must also be acknowledged is the potential for sensory PN to mask the signs and symptoms of PVD and the possibility that a proportion of those who develop this type of PN will do so with no symptoms. Whilst acknowledging the impact this would have on the reliability of determining disease prevalence using a survey alone, the inclusion of 'Doctor diagnosis' acts to reduce the likelihood of this group being undetected.

Applying 'gold standard' clinical assessment for DRFD across a widespread community based population in Australia presents many challenges. Whilst clinical examination may be the desired option for determining absolute disease prevalence, it is unfortunately a costly and time consuming exercise. Alternatives for collection of prevalence ratings for DRFD are a necessity if long term data collection is to become a reality.

## Conclusion

The Q-DFD provides a reliable alternative to clinical assessment and is strongly recommended for the ongoing collection of such data in order to fully inform health service policy and planning and assist with evaluation of current care models.

## Competing interests

The authors declare that they have no competing interests.

## Authors' contributions

SMB made a substantial contribution to the conceptual design of the study, conducted all research involving participants and undertook data collection and subsequent analysis. SMB also prepared the manuscript draft. CAB, PGC and DAC all provided methodological advice regarding the study design and provided editorial advice during preparation of the manuscript. All authors have read and approved the final manuscript.
